# Oral rhabdomyosarcoma, a rare malignant tumor mimicking an endodontic-periodontal lesion in an adult patient: a case report

**DOI:** 10.1186/s12903-024-03875-w

**Published:** 2024-01-16

**Authors:** Fang-Yu Zheng, Juan-You Qiu, Kuo-Han Liao, Nan-Chin Lin

**Affiliations:** 1 Department of Endodontics, Show Chwan Memorial Hospital, Changhua, Taiwan; 2 Department of Oral and Maxillofacial Surgery, Show Chwan Memorial Hospital, Changhua, Taiwan; 3 School of Dentistry, College of Dentistry, China Medical University, Taichung, Taiwan

**Keywords:** Embryonal rhabdomyosarcoma, Gingiva, Adult, Immunohistochemistry

## Abstract

**Background:**

According to previous research, 2.8% of lesions clinically identified as endodontic pathosis were ultimately diagnosed as non-endodontic periapical lesions via histopathology, and 3.7% of these non-endodontic periapical lesions were malignant neoplasms. Rhabdomyosarcoma, a malignant tumor most commonly observed in children, is uncommon in the oral cavity.

**Case presentation:**

This is a report of a rare case of embryonal rhabdomyosarcoma in a 41-year-old female, in which the lesion was in the maxillary gingiva. The biopsy reports confirmed the diagnosis of embryonal rhabdomyosarcoma. The wide excision of the tumor, free flap reconstruction, chemotherapy, and radiotherapy were performed. Clinical, radiological, and histopathological and management aspects of the neoplasm were also discussed.

**Conclusions:**

This case report aimed to create awareness that rhabdomyosarcoma is one of the differential diagnoses of periapical lesions.

## Background

Endodontic or periodontal problems are the most common origins of radiolucent lesions found in the jaw bone. However, according to previous research, 2.8% of lesions that are clinically identified as endodontic pathosis were ultimately diagnosed as non-endodontic periapical lesions via histopathology. And among those non-endodontic periapical lesions, 3.7% were malignant neoplasms, the most common of which was squamous cell carcinoma [1]. Other malignant diagnoses included multiple myeloma, metastatic carcinoma, and metastatic leiomyosarcoma [1]. There was another case report that also said oral squamous cell carcinoma can mimic a common endodontic-periodontal lesion, causing misinterpretation and resulting in delayed appropriate treatment [2]. Therefore, the differential diagnosis of endodontic-periodontal lesions and malignant neoplasms is critical.

In our case report, we present a rare malignant head and neck tumor, rhabdomyosarcoma mimicking an endodontic-periodontal lesion. Sarcomas are malignant neoplasms that come from mesenchymal cells. Sarcomas of the head and neck region are very rare tumors that comprise only 1% of all head and neck malignancies in adults [3]. Among the softtissue sarcomas, rhabdomyosarcoma (RMS) predominantly occurs in children (60%) and is rarely found in adults (2–5%) [4]. As for the sites of occurrence of RMS, the most frequent location is the head and neck region. RMS of the oral cavity accounts for only 10–12% of all head and neck cases [5]. RMS can be subcategorized into three subtypes including alveolar, embryonal, and pleomorphic types according to different microscopic features. Embryonal RMS is most common before the age of ten years and accounts for about 60% of all cases. Alveolar RMS often occurs between the ages of 10 years and 25 years, accounting for 20–30% of all tumors. Pleomorphic RMS accounts for less than 5% of all cases and has peak prevalence in adults older than 40 years old. RMS of the head and neck region are mostly embryonal or alveolar types, while pleomorphic types primarily occur on the extremities [6]. The early diagnosis and treatment of sarcoma are required to achieve a better prognosis. Within published research, reports of this lesion are very rare in the oral cavity in adults. Herein, we present an extremely rare case of embryonal RMS arising from the gingiva in an adult mimicking an endodontic-periodontal lesion.

## Case presentation

A 41-year-old female patient reported to the Department of Endodontics (Show Chwan Memorial Hospital, Changhua, Taiwan) with a chief complaint of a small swelling on the gingiva and spontaneous pain on her upper right front tooth that began six months earlier. The patient denied any previous traumatic event and her medical history was noncontributory. The swelling started as a small nodule and progressed slowly, associated with mild, dull intermittent non-irradiating pain. She had a history of periodontal treatment and endodontic therapy of her upper right lateral incisor at a local dental clinic. However, due to the inefficacy of the treatment, the patient was referred to our hospital for better management.

**Fig. 1:**
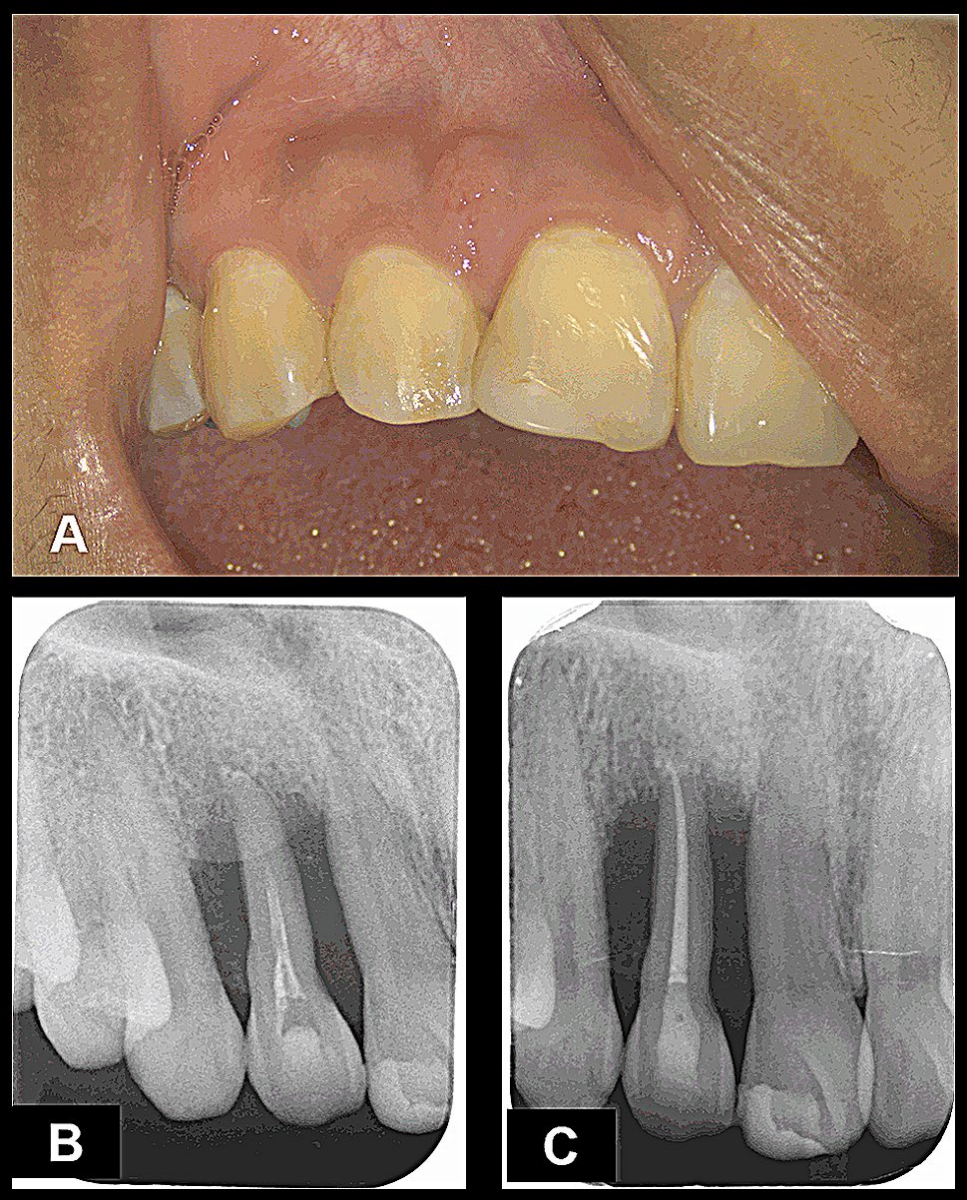
Pre-operative intraoral photograph and radiographs **(A)** The intraoral examination revealed a tender, firm, sessile growth measuring approximately 2 mm x 2 mm involving buccal attached gingivae adjacent to the maxillary right lateral incisor and central incisor. **(B)** Initial periapical radiograph, revealing aggressive bone destruction extending to the periapical area. **(C)** Periapical radiograph after endodontic retreatment performed on tooth 12

An intraoral examination revealed a tender, firm, sessile growth measuring approximately 2 mm x 2 mm, involving buccal attached gingiva adjacent to the maxillary right lateral incisor and central incisor (Fig. 1A). Her tooth 12 was caries-free, with temporary filling material at the palatal surface. Her teeth 11 and 13 responded normally to thermal and electric pulp tests, as well as to vertical and lateral percussion. Her upper right lateral incisor showed grade 1 mobility, a deep periodontal pocket measuring about 10 mm at the mesiobuccal and mesiopalatal sides, and tenderness on percussion and palpation. An intraoral radiograph revealed rarefaction of the bone with imprecise margins completely involving the root of the lateral incisor (Fig. 1B). A tentative diagnosis of an endodontic-periodontal lesion was made and the etiologies for the lesion included a palatogingival groove (PGG), root fracture, granulomatous disease, or neoplasm.

**Fig. 2:**
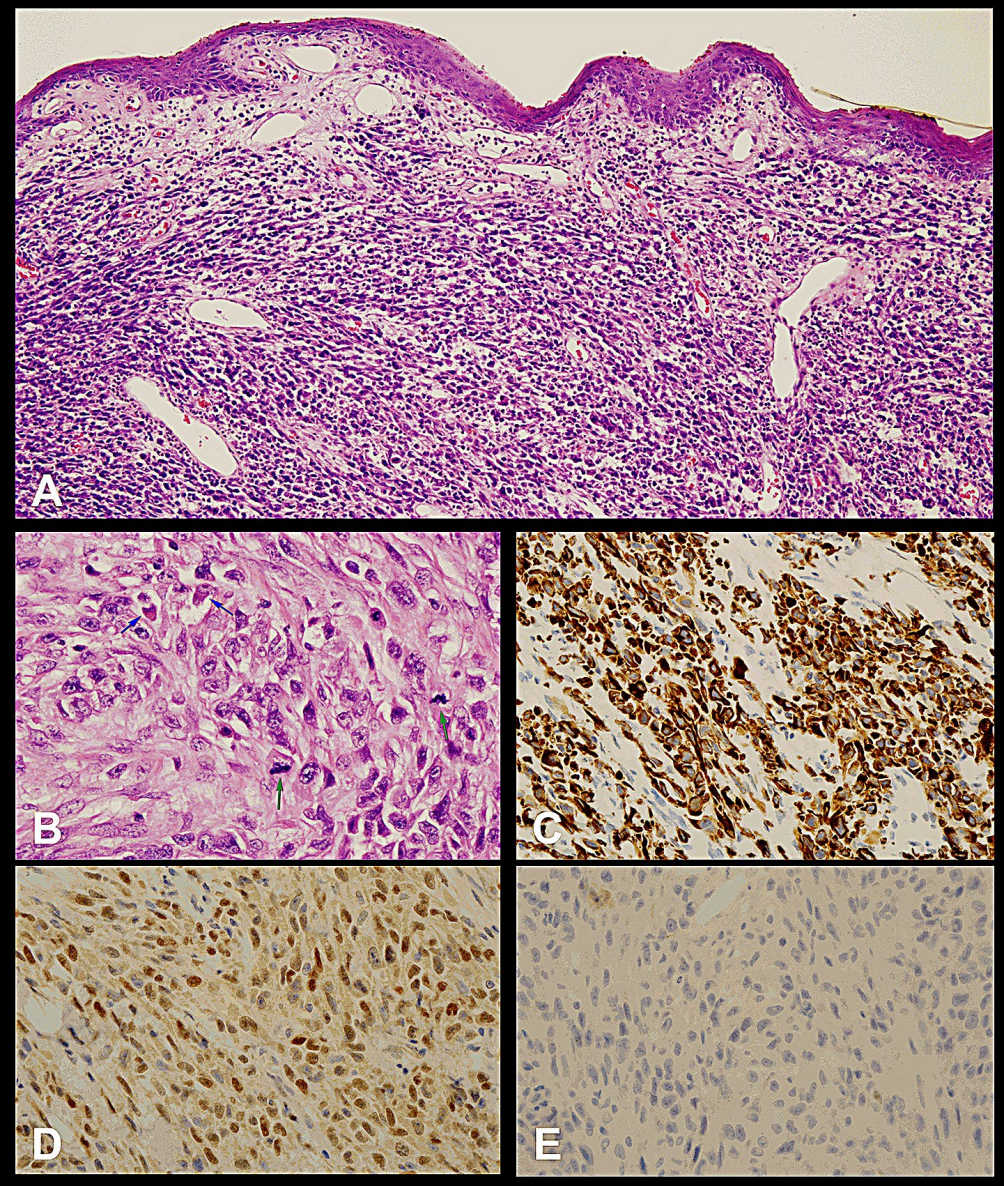
A diagnosis of embryonal rhabdomyosarcoma was given after histopathological examinations. **(A)** Low-power view demonstrating disorganized sheets of tumor cells with hypercellularity in the submucosal area and composed of spindled cells (original magnification 100 ×). **(B)** Ovoid to spindled tumor cells with abundant eosinophilic cytoplasm and eccentric prominent nucleoli and blue arrows indicating rhabdomyoblastic differentiation. Green arrows indicating mitotic tumor cells (original magnification 400 ×). **(C)** IHC stain for desmin showed diffuse and strong positive (original magnification 400 ×). **(D)** IHC stain for myoD1 showed diffuse positive (original magnification 400 ×). **(E)** IHC stain for myogenin was negative (original magnification 400 ×)

After the endodontic retreatment performed on tooth 12 (Fig. 1C), she was referred to the Department of Periodontics for further periodontal management. However, buccal and palatal tissue expansion over her maxillary right lateral incisor was noted after periodontal treatment. So, the patient was referred to an oral and maxillofacial surgeon who performed an incisional biopsy. The pathological report revealed atypical spindled tumor cells contained abundant eosinophilic cytoplasm and nuclei oval arranged in interlacing fascicles which indicating rhabdomyoblastic differentiation (Fig. 2A). Mitotic tumor cells could be found in the Fig. 2B and the immunohistochemical tests were positive for desmin and myoD1 and negative for myogenin (Fig. 2C and D, and 2E). Hence, a diagnosis of embryonal rhabdomyosarcoma was made.

**Fig. 3:**
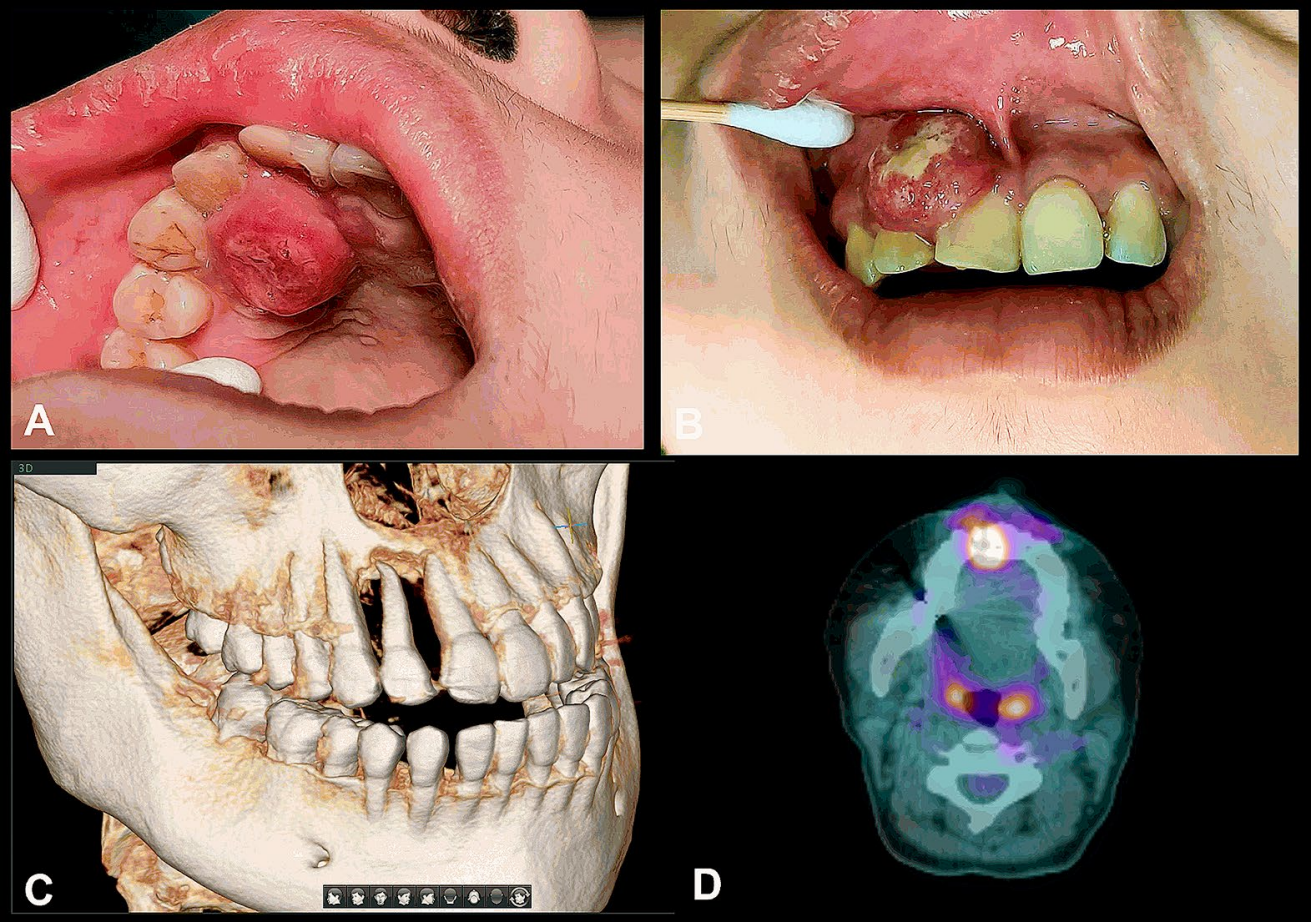
Intraoral photos and images after incisional biopsy. **(A)** Palatal view and **(B)** buccal view of the swelling, which progressed rapidly, measuring approximately 2.5 cm x 2.5 cm. The mucosa covering the lesion was reddish with grayish areas of central necrosis. **(C)** The CBCT image showed severe bony resorption around tooth 12 and irregular destruction of the maxillary bone. **(D)** PET showed a 2.6 cm high FDG-avid lesion at the upper right gingival area with right maxillary infiltration

At the recall appointment after the incisional biopsy, the swelling over buccal and palatal side progressed rapidly, measuring approximately 2.5 cm x 2.5 cm. The mucosa covering the lesion was reddish with grayish areas of central necrosis (Fig. 3A and B). Further examinations were arranged for the patient. Cone-beam computed tomography was performed to evaluate the upper right area. The buccal and palatal cortex around the upper right lateral incisor demonstrated severe erosion and an irregular destruction of the maxillary bone was noted (Fig. 3C). Contrast-enhanced MRI revealed a periapical enhancing mass at the upper right gingiva measuring 2.9 cm x 2.3 cm x 2.1 cm with suspicious invasion to the underlining maxillary bone, which is compatible with tumor growth. PET revealed a 2.6 cm high FDG-avid lesion at the upper right gingival area with right maxillary infiltration, which is compatible with a highly metabolic active malignancy (Fig. 3D). The TNM staging of cT4aN0M0 for this soft tissue sarcoma was considered.

**Fig. 4:**
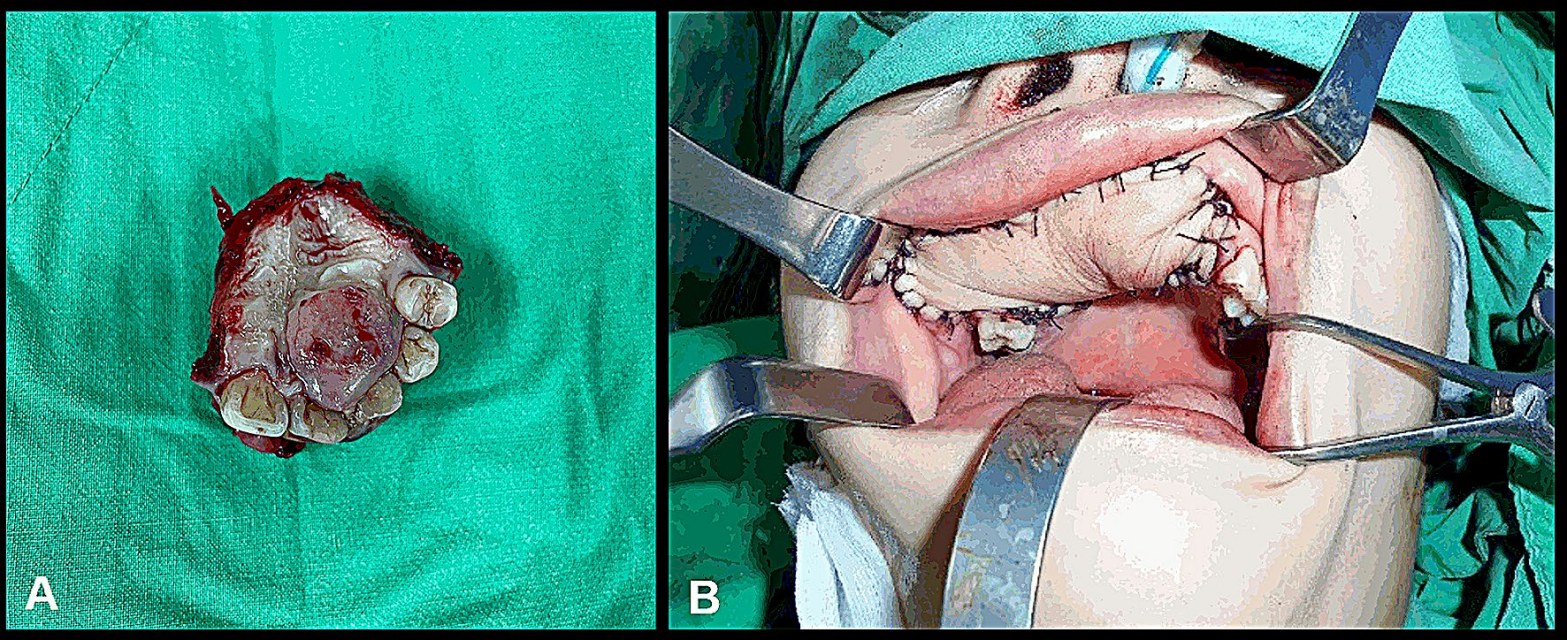
Surgical excision of the tumor and reconstruction. **(A)** The excised tissue after wide excision of the tumor. **(B)** Radial forearm free flap reconstruction

The patient was admitted to our ward, where she received further management. Our treatment plan included wide excision of the tumor and free flap reconstruction (Fig. 4). An excisional biopsy was performed during surgery, and it revealed findings similar to those of the incisional biopsy. Subsequent to surgery, she underwent radiotherapy with 60 Gy and chemotherapy with six cycles of vincristine.

## Discussion and conclusions

This case illustrates the importance of thorough clinical and radiographic evaluations for accurate diagnosis and proper treatment. A small mass over the maxillary right gingiva combined with severe bony destruction around the tooth on the intraoral radiograph was initially treated as an endodontic-periodontal lesion. After endodontic and periodontal treatment, the swelling increased; so, she was referred to an oral surgery specialist for further management. After an incisional biopsy of the lesion was performed, this lesion has been identified as embryonal rhabdomyosarcoma. From the appearance of the very first lesion to the correct diagnosis, it takes approximately four months, and diagnostic delays may lead to poor prognoses for the patient.

Rhabdomyosarcoma (RMS) is a malignant soft tissue neoplasm, which consists of cells derived from the primitive mesenchyme and exhibits a profound tendency to undergo myogenesis [7]. RMS is the most common soft tissue sarcoma in children, with an annual incidence of 4.5 cases per 1 million children [8]. In contrast, it is rare in people older than 45 years and comprises only 2–5% of soft tissue sarcomas in adults [4]. The incidence of RMS differs by age, histology, and ethnicity. In parts of Asia such as Japanese, Indian, and Chinese populations, the incidence of RMS appears to be lower than it is in Europe and the United States [9]. The common sites of occurrence are the head and neck region (35%), genitourinary tract (23%), retroperitoneum, and the extremities (17%) [4]. In the head and neck region, the commonly affected sites are the orbit, paranasal sinuses, and the neck [10]. RMS in the oral cavity is rare, accounting for only 10–12% of all head and neck cases [5]. Among cases of RMS in the oral cavity, the most frequently involved location is the tongue, followed by the palate and buccal mucosa. It may also involve the gingiva in very rare cases [4]. Therefore, the presented case of RMS arising from the gingiva in an adult is an extremely rare one.

This case illustrates the importance of thorough clinical and radiographic evaluations for accurate diagnosis and proper treatment. The clinical presentation of RMS is variable and influenced by the site, age, and the presence or absence of distant metastases [10]. The unspecific clinical characteristics pose great difficulties in the differential diagnosis when RMS appears as a common inflammatory tooth-related lesion in the oral cavity. In our case, a misdiagnosis of an endodontic-periodontal lesion was proposed based on clinical and radiographic findings. An incisional biopsy was performed since both endodontic and periodontal treatments were ineffective. An accurate diagnosis of RMS was made based on the findings of histopathological tests. Previous studies revealed that when a non-endodontic periapical lesion mimics apical pathology around a tooth, which is pulp necrosis or previously root canal treated such as in our case, the clinician may not always recognize it even after diligent clinical and radiographic evaluations [1]. Nevertheless, there are still some clues that alert the clinician to the possibility of origins other than endodontic or periodontal problems. First, Radiographically, bony destruction around tooth 12 was conical in contour with a wide base coronally and narrow at the apex of the root, suggesting a primary periodontal lesion [11]. However, periodontitis is a disease that is closely related to systemic factors; thus, the bone loss involved here is often generalized and affects multiple teeth. However, severe bone destruction only occurred around tooth 12 and the condition became even more aggravated after periodontal treatment in this case. Second, there could be many different etiologies of apical periodontitis in a given tooth. The usual causes are dental caries, trauma, cracked teeth, and re-infection of prior endodontic treatment [12]. In this case, the patient denied any trauma history. We failed to find caries and cracked lines on the surface of the tooth and the dentin wall of the canal was clear under the magnification of the dental operative microscope. Therefore, we need to consider other possible causes of the lesion from the very first. Finally, etiological factors for localized periodontal abscesses causing severe and rapid destruction of periodontal tissues were not found. According to previous studies, etiological factors of periodontal abscesses in non-periodontitis patients included subgingival calculi, foreign body impaction, harmful habits, orthodontic factors, and alterations of the root surface [13]. There was no subgingival calculus when we explored the root surface of tooth 12 with a periodontal probe after scaling and root planing. Foreign bodies such as dental floss, toothpicks, rubber dams, or food debris were not impacted in the periodontal pocket. The patient had not received orthodontic treatment, nor did she have any clenching or nail-biting habits. Anatomic alterations of the root surface such as palatal gingival grooves, cemental tears, enamel pearls, and cervical enamel projection were not found both in intraoral and radiographic examinations. Severe root damage such as root fracture, perforation, and external root resorption were not found either. Therefore, risk factors for localized aggressive periodontal destruction could be ruled out. Taken together, once a lesion of nonodontogenic origin is suspected, tissue curettage samples collected by the periodontal specialist should be sent to the pathologist for further examination.

For RMS, the initial approach for a tissue diagnosis is to get a surgical core biopsy [14]. Immunohistochemical staining plays an important role in the diagnosis of RMS. Because tumor cells of RMS often display some evidence of skeletal muscle lineage specification, desmin, myoD1, and myogenin are considered useful markers [4]. Several previous studies reported that alveolar RMS often displays robust immunohistochemical staining for myogenin, while embryonal RMS usually displays a strong response for desmin [9]. These results are consistent with the findings in our case. In other adult RMS cases, the primary lesion is generally a non-tender mass, which is different from the findings in the presented case [10]. We speculated that the pain of this patient was of dental origin, perhaps due to a previous root canal treatment and subgingival curettage. RMS occurs rarely in adults. However, all of the scant studies indicated that it is more aggressive in adults than in children, and it has a worse prognosis with a five-year overall survival rate of 26–40% [15, 16]. Hence, early diagnosis and treatment are critical. Delayed treatment leads to poor outcomes. Recently, Cavalcante et al. also reported a lower gingival rhabdomyosarcoma mimicking a reactive proliferative lesion in a 30-year-old female [17]. The tumor presented as a well-defined polypoid nodule on the mandibular gingiva clinically, and it was misdiagnosed as pyogenic granuloma. They also emphasized the importance of the careful histopathological evaluation, supported by immunohistochemistry analysis which is the key to avoid diagnostic errors [17]. Although a standard therapy is not yet established in the rare group of adult patients with RMS, many studies have suggested that a multimodal approach will achieve the best possible outcome [10]. Both radiotherapy and chemotherapy are needed. Previous studies recommend that pediatric-style chemotherapy is effective in adults. If treated with more intensive therapy, adults with RMS may have better outcomes [9]. Considering the critical role played by role of dental professionals, especially periodontists and endodontists, they should be aware that rhabdomyosarcoma may manifest itself clinically and/or radiographically as a common periodontal or endodontic lesion.

This case report aimed to create awareness that rhabdomyosarcoma is one of the differential diagnoses of periapical lesions and to illustrate the importance of thorough clinical and radiographic evaluations, as well as biopsy submission, for accurate diagnosis and treatment.

## Data Availability

The datasets used during the current study are available from the corresponding author on reasonable request.

## Abbreviations

PET: Positron Emission Tomography
RMS: Rhabdomyosarcoma
TNM: Tumor-Node-Metastasis
